# Epidemiological, Clinical and Allergic Profile of Patients With Dyshidrotic Eczema (Acute and Recurrent Vesicular Dermatitis): Evaluation of the Spanish Registry of Research in Contact Dermatitis and Cutaneous Allergy (REIDAC)

**DOI:** 10.1111/cod.70184

**Published:** 2026-05-17

**Authors:** Araceli Sánchez‐Gilo, Enrique Gómez de la Fuente, Ana M. Giménez‐Arnau, Juan Francisco Silvestre‐Salvador, Pedro Mercader‐García, Tatiana Sanz‐Sánchez, Violeta Zaragoza‐Ninet, Susana Córdoba‐Guijarro, Javier Miquel‐Miquel, Ricardo González‐Pérez, Inmaculada Ruiz‐Gonzalez, Esther Serra‐Baldrich, José Manuel Carrascosa‐Carrillo, Francisco Javier Ortiz de Frutos, Mercedes Rodríguez‐Serna, María Elena Gatica‐Ortega, Carmen Paredes‐Suárez, Francisco J. Navarro‐Triviño, Pablo Chicharro, María Antonia Pastor‐Nieto, Marta Andreu Barasoain, José‐Juan Pereyra‐Rodríguez, Gemma Melé‐Ninot, Paloma Sánchez‐Pedreño Guillén, Marta Elosua‐González, Miguel Ángel Descalzo, Ignacio García‐Doval, Raquel Barba‐Martín, Leopoldo Borrego

**Affiliations:** ^1^ Escuela Internacional de Doctorado, Rey Juan Carlos University Madrid Spain; ^2^ Department of Dermatology Hospital Universitario Rey Juan Carlos Madrid Spain; ^3^ Department of Dermatology Hospital Ramón y Cajal Madrid Spain; ^4^ Department of Dermatology Hospital del Mar Research Institute Barcelona Spain; ^5^ Universitat Pompeu Fabra Barcelona Spain; ^6^ Department of Dermatology Hospital General Universitario Dr Balmis. ISABIAL Alicante Spain; ^7^ Department of Dermatology Hospital General Universitario José María Morales Meseguer Murcia Spain; ^8^ Department of Dermatology Hospital Universitario Infanta Sofía Madrid Spain; ^9^ Departamento de Medicina Facultad de Medicina Salud y Deporte, Universidad Europea de Madrid Madrid Spain; ^10^ Department of Dermatology Hospital General Universitario de Valencia Valencia Spain; ^11^ Department of Dermatology Hospital Universitario de Fuenlabrada Madrid Spain; ^12^ Department of Dermatology Hospital Arnau de Vilanova de Valencia Valencia Spain; ^13^ Department of Dermatology Hospital Universitario Araba, Universidad del País Vasco Vitoria‐Gasteiz Spain; ^14^ Department of Dermatology Complejo Asistencial Universitario de León (CAULE) León Spain; ^15^ Department of Dermatology Hospital de la Santa Creu i Sant Pau Barcelona Spain; ^16^ Department of Dermatology Hospital Universitario Germans Trias i Pujol Badalona Spain; ^17^ Department of Dermatology Hospital 12 de Octubre Madrid Spain; ^18^ Department of Dermatology Hospital de la Fe Valencia Spain; ^19^ Department of Dermatology Complejo Hospitalario Universitario de Toledo Toledo Spain; ^20^ Department of Dermatology Complejo Hospitalario Universitario de Santiago Santiago de Compostela Spain; ^21^ Department of Dermatology Hospital Universitario San Cecilio Granada Spain; ^22^ Department of Dermatology Hospital Universitario de la Princesa Madrid Spain; ^23^ Department of Dermatology Hospital Universitario de Guadalajara Guadalajara Spain; ^24^ Department of Dermatology Hospital Fundación Jiménez‐Díaz Madrid Spain; ^25^ Department of Dermatology Hospital Universitario Fundación Alcorcón Madrid Spain; ^26^ Department of Dermatology Hospital Universitario Virgen del Rocío Sevilla Spain; ^27^ Department of Medicine, Faculty of Medicine University of Seville Seville Spain; ^28^ Department of Dermatology, Hospital Universitari Sagrat Cor, Grupo Quirónsalud Barcelona Spain; ^29^ Department of Dermatology Hospital Clínico Universitario Virgen de la Arrixaca Murcia Spain; ^30^ Department of Dermatology Hospital Universitario Puerta de Hierro Madrid Spain; ^31^ Unidad de Investigación Academia Española de Dermatología y Venereología Madrid Spain; ^32^ Department of Dermatology Complexo Hospitalario Universitario de Vigo Vigo Spain; ^33^ Department of Internal Medicine Hospital Universitario Rey Juan Carlos Madrid Spain; ^34^ Department of Dermatology Hospital Universitario Insular de Gran Canaria Las Palmas de Gran Canaria Spain; ^35^ Universidad de Las Palmas de Gran Canaria Las Palmas de Gran Canaria Spain

**Keywords:** acute and recurrent vesicular dermatitis, dyshidrotic eczema, epidemiological surveillance, hand eczema, patch testing, pompholyx

## Abstract

**Background:**

Dyshidrotic eczema (DE), also termed acute and recurrent vesicular dermatitis, is a vesicular clinical pattern of hand eczema (HE) with heterogeneous aetiologies.

**Objectives:**

To characterise the clinical‐allergological profile of patients with DE and to compare it with non‐DE HE subtypes.

**Methods:**

A multicentre, retrospective, observational study was conducted using data from the Spanish Contact Dermatitis Registry (2019–2024).

**Results:**

Of 4378 patients with HE, 559 were diagnosed with DE. A longer median disease duration (24 vs. 14 months) and more frequent palmoplantar involvement (16% vs. 7%) were observed in DE compared with non‐DE HE. At least one positive patch test was identified in 43% of DE versus 52% in non‐DE HE. Although overall sensitisation patterns were largely comparable, DE showed lower sensitisation rates to 2‐HEMA, carba mix and thiuram mix. Occupational factors were less frequently implicated in DE (9% vs. 29%) compared with non‐DE HE.

**Conclusions:**

Despite a high overall frequency of contact allergen sensitisation, the pattern of findings suggests that the dyshidrotic phenotype may follow a clinical course less dependent on external or occupational triggers, pointing towards a relatively greater contribution of endogenous factors compared with other HE subtypes.

## Graphical abstract

In this exploratory analysis, dyshidrotic eczema demonstrated a distinct sensitisation pattern compared with non‐dyshidrotic hand eczema. The lower proportion of clinically relevant reactions amongst positive patch tests suggests a multi‐factorial and not exclusively allergic background.

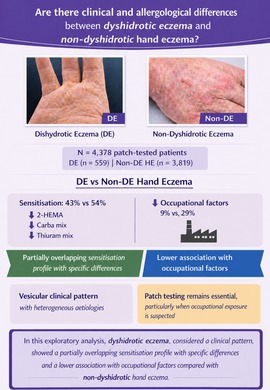

## 1 Introduction

Traditionally, dyshidrotic eczema (DE) has been considered a clinical subtype of hand eczema (HE), characterised by the appearance of intensely pruritic vesicles on the lateral aspects of the fingers and the palms, which tend to resolve spontaneously within 2–3 weeks, with a tendency to recur. In subacute and chronic phases, it may be associated with erythema, scaling and fissuring [1, 2]. Over time, DE has been regarded as a reactive pattern present in the hands and/or feet common to different aetiologies, including allergic and irritant contact dermatitis, atopic dermatitis, systemic contact dermatitis, fungal infections and id reactions (autoeczematization secondary to a distant infectious focus, most commonly due to dermatophyte infection e.g., tinea pedis); however, in a significant proportion of patients, no specific cause can be identified [1, 3, 4, 5].

Several terms have historically been used to describe this condition, including dyshidrosis, pompholyx and acute and recurrent vesicular dermatitis (ARVD) [2]. In contemporary clinical practise and in clinical registries such as the Spanish Registry of Research in Contact Dermatitis and Cutaneous Allergy (REIDAC), the term DE is commonly applied to describe a recurrent vesicular phenotype, regardless of the final etiological diagnosis established after diagnostic evaluation. Although the term ARVD may be conceptually more precise, the term DE is used throughout the manuscript for consistency with the terminology used in the REIDAC registry [6]. The use of different terms to define the same entity, changes in the classification of chronic HE (CHE), and heterogeneity in study designs have resulted in substantial variability in published results, thereby hindering comparative analysis.

Large multi‐centre studies characterising the clinical and allergological profile of DE are scarce, and not all studies performed patch testing in all patients. Most published series are smaller [1], whilst other studies are old, including earlier series such as Meneghini and Angelini [7], or focus on alternative outcomes, such as quality of life or economic impact, rather than a systematic allergological assessment [8, 9] (Table S1). In this context, a multicentre clinical study was designed to describe the epidemiological, clinical and allergological profile of patients with a clinical diagnosis of hand DE, evaluated by patch testing, and to compare them with other subtypes of HE with non‐dyshidrotic morphology (non‐DE HE).

## 2 Methods

### 2.1 Study Design

We conducted a retrospective, observational, multi‐centre, descriptive study, using the REIDAC registry, with exploratory comparative analyses. This manuscript was prepared in accordance with the STROBE (Strengthening the Reporting of Observational Studies in Epidemiology) reporting guidelines. The completed STROBE checklist is provided as Supporting Information to support transparent and complete reporting (Table S2).

### 2.2 Data Source and Study Period

Study data were collected and managed using REDCap electronic data capture tools (http://www.project‐redcap.org/; RRID:SCR_003445) hosted at the Fundación Piel Sana. The REIDAC currently collects standardised information on patients undergoing patch testing in 24 Spanish centres. The registry involves dermatologists with expertise in contact dermatitis, and reflects real‐world clinical practise [10]. The clinical data are the same and belong to the same categories as those defined in the ESSCA minimal data sheet [11].

Patch testing was performed according to ESCD recommendations, with readings on D2/D3 and D4/D7 in accordance with the protocol. Allergen concentrations and vehicles corresponded to those established in the Spanish baseline series and extended baseline series [12]. Clinical relevance was considered present according to the information recorded in the medical history [13].

In the REIDAC, both a main and a secondary diagnosis can be recorded. There is no specific category for “hand eczema”; however, “hands” is defined as a primary anatomical site. Amongst the diagnoses available in the registry are both aetiological and morphological categories, such as allergic contact dermatitis (ACD), irritant contact dermatitis (ICD), atopic dermatitis (AD), protein contact dermatitis, nummular eczema and DE, established according to the clinician's final judgement after diagnostic evaluation [11].

For the present study, collected variables were age, sex, atopic background (atopic dermatitis, asthma, allergic rhinoconjunctivitis), duration of symptoms, plantar involvement, concomitant diagnoses, occupation and occupational factors, patch test results (baseline series and extended baseline series) and clinical relevance.

### 2.3 Inclusion and Exclusion Criteria

The inclusion period extended from 1 January 2019 to 31 December 2024. All consecutive patients referred for patch testing with a recorded diagnosis of DE as a primary or secondary diagnosis were included (DE group), with or without plantar involvement. These patients were descriptively compared with those with other subtypes of HE in whom DE was not selected, according to the final diagnostic classification established by the clinician after evaluation (non‐DE HE group). Cases with exclusive plantar involvement without hand lesions were excluded.

### 2.4 Operational Definitions

*Dyshidrotic eczema (DE):* In the REIDAC, the final diagnosis is established after a comprehensive clinical evaluation including lesion morphology, clinical course and patch test results. DE is used to describe a recurrent vesicular clinical pattern affecting the hands and/or feet, which may be associated with one or more final aetiological diagnoses, according to the evaluator's clinical judgement. REIDAC does not systematically capture longitudinal disease patterns, as data are collected at a single time point and classification is based on clinical judgement at the time of evaluation. Therefore, disease course and remission phases between flares cannot be reliably assessed.

*Non‐DE HE:* hand eczema in which vesicular morphology is not specified in the diagnosis.

*Sensitisation:* Presence of at least one positive reaction in the Spanish baseline series and extended baseline series of the Spanish Research Group on Contact Dermatitis and Cutaneous Allergy (GEIDAC) [12]. Patch test reactions scored as +, ++ or +++ were considered positive, whilst doubtful or irritant reactions were considered negative for the analysis.

*Disease duration:* Disease duration was defined as the interval between self‐reported symptom onset and the time of patch testing.

### 2.5 Statistical Analysis

A descriptive analysis of clinical, epidemiological and allergological variables was performed. Quantitative variables were expressed as mean (standard deviation) or median (interquartile range), according to their distribution, whilst categorical variables were expressed as frequencies and percentages. Comparisons between the DE and non‐DE HE groups were conducted using bivariate tests: Student's *t*‐test or Mann–Whitney *U* test for continuous variables, and Pearson's *χ* ^2^ test or Fisher's exact test for categorical variables, as appropriate. For allergen sensitisation analyses, prevalences were calculated and, exploratorily, prevalence ratios (PRs) with 95% confidence intervals (95% CIs) were derived from 2 × 2 tables. Given the exploratory nature of the analysis, *p*‐values are presented for descriptive purposes only and should not be interpreted as confirmatory evidence. All analyses were carried out using STATA v.19.0 (Stata Corp. 2025).

### 2.6 Ethical Considerations

The study was approved by the Research Ethics Committee of the Insular–Materno Infantil University Hospital Complex of Las Palmas de Gran Canaria (approval no. 2017/964, 2 November 2017). All data were analysed in anonymised form in accordance with current regulations, and signed informed consent for inclusion in the project was obtained from all patients.

The REIDAC was approved by the Research Ethics Committee of the Complejo Hospitalario Universitario Insular‐Materno Infantil (CEIm‐CHUIMI‐2017/964). Written informed consent was obtained from all participants prior to their inclusion in the study.

### 2.7 Funding Information

The Spanish Registry of Contact Dermatitis (REIDAC) is supported by the Fundación Piel Sana, of the Spanish Academy of Dermatology and Venereology (AEDV), and receives non‐conditioned funding from both the Spanish Agency for Medicines and Health Products and from pharmaceutical companies (Sanofi and LeoPharma, previously GlaxoSmithKline and Novartis). None of the funding sources or sponsors had any role in the study design, data collection, analysis, interpretation, manuscript preparation or the decision to submit the work for publication.

## 3 Results

During the study period (1 January 2019 to 31 December 2024), a total of 4378 patients patch tested for HE were recorded in the REIDAC. Of these, 559 patients (12.8%) were classified as having DE and 3819 (87.2%) as non‐DE HE. The demographic and clinical characteristics of both groups are shown in Table 1. Aetiology and concomitant diseases according to the REIDAC registry are shown in Table 2.

**TABLE 1:** Clinical and epidemiological characteristics of patients with DE group^a^ versus non‐DE hand eczema^b^.

Variable	DE *N* (%)	Non‐DE hand eczema *N* (%)	*p*
Total patch‐tested patients	559 (100)	3819 (100)	
Female sex	363 (65)	2628 (69)	0.0658
Age, mean (SD)	41.6 (15.4)	43.9 (15.6)	0.0012
Age > 40 years	300 (54)	2263 (59)	0.0133
Symptom duration, months (median, IQR)	24.0 (12–48)	14.0 (10–36)	< 0.0001
History of atopic dermatitis	90 (16)	610 (16)	0.9670
Asthma	71 (13)	386 (10)	0.0636
Allergic rhinoconjunctivitis	148 (27)	785 (21)	0.0017
Foot involvement	89 (16)	269 (7)	< 0.0001
≥ 1 positive patch test (baseline series)	229 (41)	1979 (49)	< 0.0001
≥ 1 positive (baseline + extended series)	243 (43)	1979 (52)	0.0002
Occupational factors	47 (9)	1063 (29)	< 0.0001

*Note:* Data are expressed as number of patients (percentage), unless otherwise indicated.

Abbreviations: IQR, interquartile range; SD, standard deviation.

^a^ DE: dyshidrotic ezcema.

^b^ Non‐DE: eczema.

**TABLE 2:** Aetiology and associated diagnoses in patients with DE^a^ according to the REIDAC registry.

Category	Diagnosis	*N* (%)
Aetiology	Allergic contact dermatitis	110 (19.7)
	Irritant contact dermatitis	69 (12.3)
	Atopic dermatitis	37 (6.6)
	Protein contact dermatitis	0 (0.0)
	No specific aetiology	343 (61.4)
Associated diagnoses	Seborrhoeic dermatitis	7 (1.3)
	Psoriasis	3 (0.5)
	Nummular eczema	3 (0.5)
	Non‐contact urticaria	2 (0.4)
	Exanthems	1 (0.2)
	Lichen planus	1 (0.2)
	Pruritus sine materia	1 (0.2)
	Other	4 (0.7)

^a^ DE: dyshidrotic eczema.

Several variables differed between groups, with younger age, longer disease duration, plantar involvement and allergic rhinoconjunctivitis more frequently observed in patients with DE than in non‐DE HE. Sensitization and occupational factors were less frequent in DE than in non‐DE HE. Asthma and sex showed similar distributions between groups, although asthma was numerically more frequent in the DE group. In 61.4% of DE cases, no specific aetiology could be identified after diagnostic evaluation.

Age distribution within the DE group is shown in Table S3.

Regarding the sensitisation profile, 43% of patients with DE showed at least one positive reaction to the Spanish baseline series and extended baseline series, compared with 52% in the non‐DE HE group. The most frequent allergens in the DE group were nickel sulphate, methylisothiazolinone (MI), cobalt chloride, propolis, fragrance mix I, methyldibromoglutaronitrile and 2‐hydroxyethyl methacrylate (2‐HEMA), followed by balsam of Peru, potassium dichromate and *p*‐phenylenediamine. Amongst sensitised patients, MI and 2‐HEMA showed the highest current clinical relevance (74% and 75%, respectively), whereas nickel sulphate was mainly associated with past relevance. Comparative analysis revealed a distinctive profile in the dyshidrotic phenotype, characterised by a lower frequency of sensitisation to 2‐HEMA, carba mix, thiuram mix, benzisothiazolinone and hydroperoxides of linalool at 0.5%. Hydroxyisohexyl 3‐cyclohexene carboxaldehyde (Lyral) was more frequently observed in DE (Tables 3 and S4).

**TABLE 3:** Prevalence of allergic sensitisation according to diagnosis in patients with DE^a^ group versus non‐DE hand eczema^b^. Spanish baseline series 2022 and extended baseline series 2024.

Allergen (concentration, vehicle)	DE positive *N* (%)	Non‐DE hand eczema positive *N* (%)	PR	95% CI
Nickel sulphate 5.0% petrolatum	135 (24.15)	881 (23.14)	1.04	[0.89, 1.22]
Wool alcohols (lanolin) 30.0% petrolatum	3 (0.54)	14 (0.37)	1.46	[0.42, 5.06]
Neomycin sulphate 20.0% petrolatum	1 (0.18)	22 (0.58)	0.31	[0.04, 2.29]
Potassium dichromate 0.5% petrolatum	18 (3.23)	143 (3.76)	0.86	[0.53, 1.39]
Caine mix 10.0% petrolatum	3 (0.65)	18 (0.59)	1.10	[0.32, 3.72]
Fragrance mix I 8.0% petrolatum	22 (3.94)	151 (3.97)	0.99	[0.64, 1.54]
Colophony 20.0% petrolatum	5 (0.89)	48 (1.26)	0.71	[0.28, 1.78]
Paraben mix 16.0% petrolatum	3 (0.54)	9 (0.24)	2.27	[0.62, 8.37]
Balsam of Peru 25.0% petrolatum	18 (3.22)	121 (3.18)	1.01	[0.62, 1.65]
Cobalt chloride 1.0% petrolatum	30 (5.37)	185 (4.86)	1.10	[0.76, 1.61]
*p*‐tert‐Butylphenol formaldehyde resin 1.0% petrolatum	4 (0.72)	41 (1.08)	0.66	[0.24, 1.85]
Epoxy resin 1.0% petrolatum	4 (0.72)	34 (0.89)	0.80	[0.29, 2.25]
Carba mix 3.0% petrolatum	3 (0.54)	116 (3.06)	0.18	[0.06, 0.55]
IPPD/black rubber mix 0.1% petrolatum	3 (0.54)	26 (0.68)	0.79	[0.24, 2.59]
MCI/MI 0.02% aqueous	21 (4.61)	170 (5.45)	0.84	[0.54, 1.31]
Quaternium‐15 1.0% petrolatum	7 (1.25)	24 (0.63)	1.99	[0.86, 4.59]
*p*‐Phenylenediamine 1.0% petrolatum	15 (2.68)	114 (2.99)	0.90	[0.53, 1.52]
Formaldehyde 2.0% aqueous	8 (1.71)	79 (2.44)	0.70	[0.34, 1.44]
Mercapto mix 2.0% petrolatum	1 (0.18)	13 (0.34)	0.52	[0.07, 4.00]
Thiuram mix 1.0% petrolatum	3 (0.54)	105 (2.76)	0.19	[0.06, 0.61]
Diazolidinyl urea (Germall II) 2.0% petrolatum	2 (0.36)	13 (0.34)	1.05	[0.24, 4.63]
Tixocortol‐21‐pivalate 0.1% petrolatum	2 (0.36)	7 (0.18)	1.95	[0.41, 9.36]
Imidazolidinyl urea (Germall 115) 2.0% petrolatum	1 (0.18)	12 (0.32)	0.57	[0.07, 4.36]
Budesonide 0.01% petrolatum	1 (0.18)	15 (0.39)	0.45	[0.06, 3.43]
Mercaptobenzothiazole 2.0% petrolatum	2 (0.36)	16 (0.42)	0.85	[0.20, 3.69]
Methylisothiazolinone 0.2% aqueous	39 (7.72)	274 (8.14)	0.95	[0.69, 1.31]
Fragrance mix II 14.0% petrolatum	10 (1.98)	100 (2.97)	0.67	[0.35, 1.27]
2‐Hydroxyethyl methacrylate (2‐HEMA) 2.0% petrolatum	12 (3.01)	302 (11.37)	0.26	[0.15, 0.47]
Textile dye mix 6.6% petrolatum	4 (1.02)	65 (2.48)	0.41	[0.15, 1.12]
Hydroperoxides of linalool 1.0% petrolatum	10 (2.49)	122 (4.58)	0.54	[0.29, 1.03]
Hydroperoxides of limonene 0.3% petrolatum	9 (2.23)	97 (3.63)	0.61	[0.31, 1.20]
Methyldibromo glutaronitrile 0.1% petrolatum	12 (3.63)	88 (3.74)	0.97	[0.54, 1.75]
Sesquiterpene lactone mix 0.1% petrolatum	0 (0.00)	11 (0.40)	NA	NA
Hydroxyisohexyl 3‐cyclohexene carboxaldehyde (Lyral) 5% petrolatum	6 (1.46)	11 (0.40)	3.61	[1.34, 9.72]
Propolis 10% petrolatum	12 (4.55)	109 (6.05)	0.75	[0.42, 1.34]
Sodium disulfite 1% petrolatum	4 (1.52)	35 (1.94)	0.78	[0.28, 2.18]
2‐Bromo‐2‐nitropropane‐1,3‐diol (Bronopol) 0.5% petrolatum	0 (0.00)	12 (0.67)	NA	NA
Compositae mix II 5% petrolatum	0 (0.00)	6 (0.93)	NA	NA
Benzisothiazolinone 0.1% petrolatum	6 (2.27)	110 (6.08)	0.37	[0.17, 0.84]
Octylisothiazolinone 0.1% petrolatum	2 (0.76)	19 (1.05)	0.72	[0.17, 3.07]
Decyl glucoside 5% petrolatum	1 (0.38)	6 (0.33)	1.14	[0.14, 9.42]
Sorbitan sesquioleate 20% petrolatum	0 (0.00)	4 (0.57)	NA	NA
Sorbitan monooleate (Span 80) 5% petrolatum	0 (0.00)	2 (0.29)	NA	NA
Hydroperoxides of linalool 0.5% petrolatum	5 (1.25)	84 (3.21)	0.39	[0.16, 0.95]
Hydroperoxides of limonene 0.2% petrolatum	4 (1.01)	65 (2.50)	0.40	[0.15, 1.10]

*Note:* PRs below 1 indicate a lower prevalence of sensitisation in patients with DE compared with those with non‐DE hand eczema. Confidence intervals are presented for descriptive purposes.

Abbreviations: CI: confidence intervals; PR: prevalence ratios.

^a^ DE: dyshidrotic ezcema.

^b^ Non‐DE: eczema.

**FIGURE 1:**
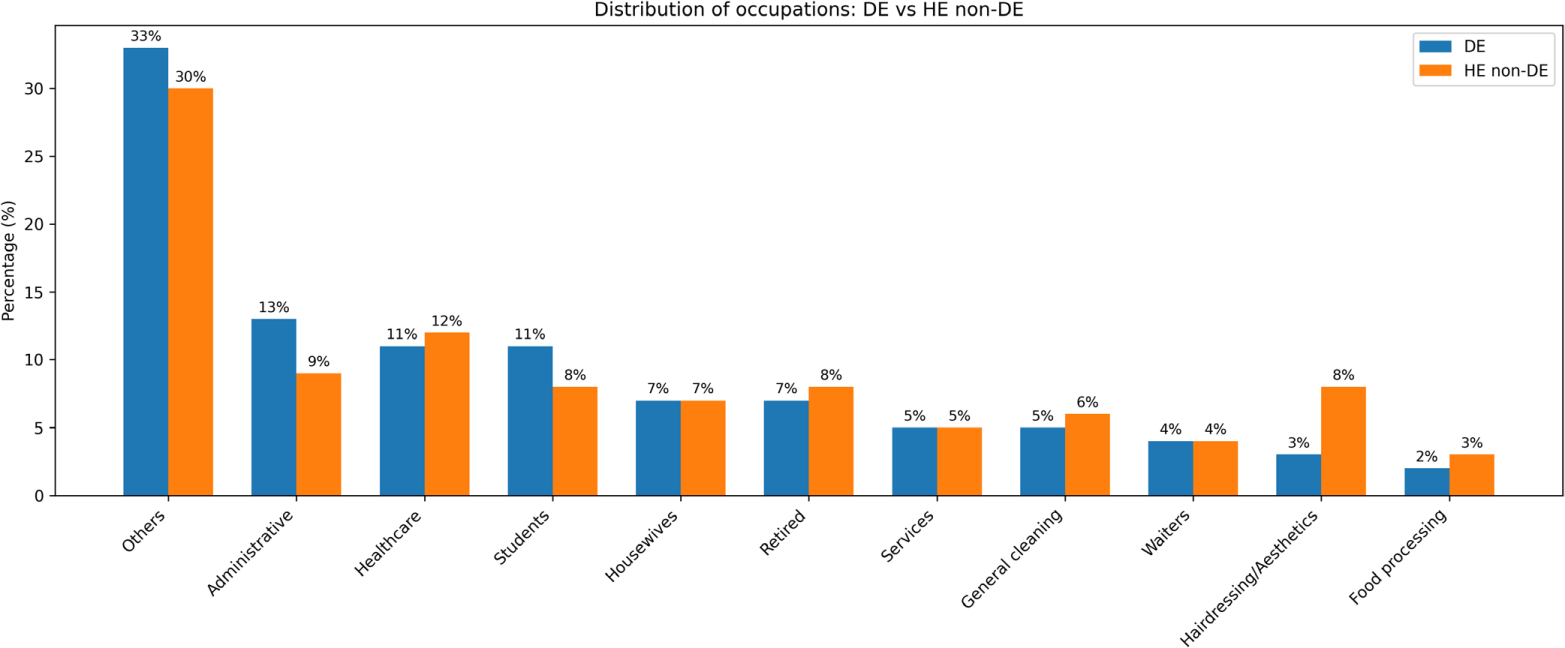
Comparative distribution of occupational categories between patients with dyshidrotic eczema (DE) and hand eczema without dyshidrotic eczema (Non‐DE HE). “Others” includes occupations representing < 3% of the dataset (low‐frequency occupations).

Occupational factors were present in 9% of patients with DE, compared with 29% of those with non‐DE HE. In the DE group, the most frequent occupations were administrative workers, healthcare workers and students. Exploratory analysis showed that administrative workers and students were more frequently observed in the DE group, whereas hairdressing/beauty professions were less frequent. The other occupational categories showed no clear differences between groups (Table S5, Figure 1).

## 4 Discussion

This multi‐centre study based on the REIDAC characterises the epidemiological, clinical and allergological profile of DE in specialised contact dermatitis units. To our knowledge, it represents the largest observational series to date specifically addressing this question in a population in which all patients underwent patch testing. Although one published study includes a larger sample size, it focused on economic outcomes and did not include systematic allergological assessment [9].

During the study period, a total of 4378 patients with HE undergoing patch testing were recorded in REIDAC, of whom 559 (12.8%) patients were classified as having DE. This prevalence falls within the range reported in the literature (5%–20%), although higher rates have been described in selected populations, likely reflecting methodological and referral heterogeneity [14, 15].

Several variables differed between groups. Younger age, longer disease duration, plantar involvement and allergic rhinoconjunctivitis were more frequently observed in DE, whereas contact sensitisation and occupational factors were more frequent in non‐DE HE. Sex and asthma showed comparable distributions between groups.

These findings reflect real‐world clinical practise, where the final diagnosis is established through an integrated assessment of morphology, clinical course and patch test results. Within this framework, DE appears best understood as a clinical pattern associated with a heterogeneous clinical and allergological profile, potentially overlapping with different aetiological subtypes of HE. Notably, in 61.4% of cases no specific aetiology could be identified after diagnostic evaluation. This proportion exceeds that reported in previous series, including Guillet et al. (15%) and other observational studies (up to 30%) [1, 16], and may reflect both a true endogenous component and limitations in current diagnostic approaches.

### 4.1 Clinical Characteristics

The demographic profile observed in our study population is consistent with previous reports, with DE predominantly affecting middle‐aged adults [1, 17]. Sex distribution showed minimal differences compared with other forms of hand eczema [5, 9, 18, 19, 20, 21, 22]. In our registry‐based population, 16% of patients with DE had a history of AD, 27% allergic rhinoconjunctivitis and 13% asthma. Although DE has traditionally been linked to atopy and proposed as a variant of ad [1, 15, 23, 24, 25], this relationship remains inconsistent across studies [18, 26, 27]. In our series, only allergic rhinoconjunctivitis was more frequently observed in DE (27% vs. 21%), whilst other atopic variables showed similar distributions.

Plantar involvement was more frequent in DE than in non‐DE HE (16% vs. 7%; *p* < 0.0001). Although DE primarily affects the hands, palmoplantar involvement has been reported in 20%–30% of cases, with around 10% showing exclusive plantar involvement [1, 28]. Our findings are consistent with previous studies reporting rates of 17.9%–20%, and lower than those described in some series (30%–46%) [1, 23, 28]. This may reflect the clinical spectrum of DE, which often includes vesicular involvement of both hands and feet. Although some studies have suggested that plantar involvement in HE may be associated with greater disease severity; however, this could not be formally assessed in our study because severity scales were not measured [28].

All cases met criteria for CHE, with longer time from symptom onset to patch testing (disease duration) than non‐DE HE, with a median disease duration of 24 months (IQR 12–48), longer than in non‐DE HE (14 months) and similar to previous studies [19]. This delay in patch testing may reflect a tendency to interpret DE within the context of AD or to assume a non‐allergic aetiology, potentially delaying further investigations. Nevertheless, the prevalence of at least one positive patch test reaction was 43% in DE versus 52% in non‐DE HE (*p* < 0.005), underscoring the importance of patch testing in these patients [1, 27].

### 4.2 Pacht Test Profile and Clinical Relevance

DE showed a high frequency and heterogeneity of contact allergen sensitisation in this selected REIDAC population, supporting the clinical value of identifying relevant sensitisations to optimise individualised management. Sensitisation rates were within the range reported in previous studies (30%–52%), with higher rates in selected populations [7, 19, 23, 29]. Patients in this study were evaluated in specialised units; therefore, sensitisation rates are expected to be higher than in the general population.

The most frequent allergens were metals, followed by isothiazolinones, propolis, fragrances, methyldibromoglutaronitrile and 2‐hydroxyethyl methacrylate (2‐HEMA) and *p*‐phenylenediamine, in line with published data [18, 19, 23, 29, 30]. Notably, 2‐HEMA sensitisation (3.0%) likely reflects the increasing exposure to acrylates, particularly in nail cosmetics [31].

The prevalence of propolis sensitisation was low and clinical relevance limited, suggesting that it may represent a potential emerging environmental factor in selected patients, although further investigation is required.

Lower frequencies of sensitisation were observed in DE for several allergens, including 2‐HEMA, carba mix, thiuram mix and benzisothiazolinone. In our series, these allergens were more frequent in non‐DE HE, which may reflect allergens more typically related to ACD, often occupational, and more frequent in non‐DE HE. Importantly, although 43% of patients with DE showed at least one positive patch test reaction, only 20% were ultimately diagnosed with ACD, as clinical relevance is required to establish causality. Overall, this pattern suggests that, in a proportion of cases, the dyshidrotic phenotype may be less dependent on external triggers and may involve a relatively greater endogenous component. Our findings support the interpretation of DE as a morphological pattern rather than a distinct aetiological entity, aligning with current conceptual frameworks in hand eczema classification.

Differences were observed for hydroperoxides of linalool 0.5%, which were more frequent in non‐DE HE, and for hydroxyisohexyl 3‐cyclohexene carboxaldehyde (Lyral), which was more frequent in the DE group. These findings should be interpreted with caution, as consistent patterns were not observed across related allergens, including hydroperoxides of linalool 1.0% and fragrance mix II.

Clinical relevance analysis showed that methylisothiazolinone and 2‐HEMA had the highest rates of current relevance in the DE group (74% and 75%, respectively), with high relevance also observed for hydroxyisohexyl 3‐cyclohexene carboxaldehyde (Lyral) (83%, *n* = 5). Nickel predominantly showed past relevance (57.8%), whilst 27.4% showed current relevance. This pattern is consistent with nickel being a ubiquitous allergen with frequent historical sensitisation, although it may still contribute to current disease activity in selected cases.

Reported relevance rates in the literature range from 59.5% to 67.5%. Allergens with higher clinical relevance typically include cosmetics, metals, fragrances, rubbers and *p*‐phenylenediamine (PPDA), several of which—such as MI and 2‐HEMA—are related to manual handling of products or glove use, sometimes in occupational settings [1, 19].

### 4.3 Occupational Factors

Occupational factors were identified in 9% of patients with DE compared with 29% in non‐DE HE, lower than previously reported (30.5%) [18]. These differences may reflect variations in the population studied. The most frequent occupations were administrative workers and students, consistent with previous reports, although distributions may vary geographically [18, 19]. Exploratory analysis showed that administrative workers and students were more frequently observed in the DE group, whereas hairdressing/beauty professions were less frequent. These findings reflect a pattern within our sample and should not be interpreted as population‐level risks.

### 4.4 Limitations

This study has limitations inherent to its retrospective observational design and multi‐centre registry‐based approach, including potential selection bias due to inclusion of patients from specialised units undergoing patch testing. In REIDAC, the diagnosis of DE is established through an integrative clinical approach incorporating morphology, disease course and patch test results. Whilst this reflects real‐world practise, it may introduce diagnostic overlap and classification challenges between morphological patterns and final aetiologies. Nevertheless, the large sample size, multicentre design and use of a standardised national registry represent key strengths, supporting the external validity of the findings within specialised clinical settings.

## 5 Conclusions

Our data show a low frequency of occupational factors and a high proportion of patients without an identifiable associated diagnosis, supporting a possible endogenous contribution in DE. Current classifications define DE as a clinical rather than an aetiological subtype, best described by the term ARVD. Establishing a clear and consistent morphological definition of dyshidrotic HE would help harmonise future studies and enable more robust comparisons. Importantly, a dyshidrosiform morphology does not exclude clinically relevant sensitisation, supporting the interpretation of DE as a vesicular morphological pattern rather than a distinct aetiological entity. This perspective supports individualised management, including patch testing when clinically indicated, particularly in recurrent or refractory cases. These findings should be interpreted within specialised settings, where sensitisation rates are expected to be higher than in the general population.

## Author Contributions

**Araceli Sánchez‐Gilo:** conceptualization, methodology, data curation, investigation, project administration, visualization, writing – original draft. **Enrique Gómez de la Fuente:** investigation, writing – review and editing, methodology, project administration. **Ana M. Giménez‐Arnau:** investigation, writing – review and editing. **Juan Francisco Silvestre‐Salvador:** investigation, writing – review and editing. **Pedro Mercader‐García:** investigation, writing – review and editing. **Tatiana Sanz‐Sánchez:** investigation, writing – review and editing. **Violeta Zaragoza‐Ninet:** investigation, writing – review and editing. **Susana Córdoba‐Guijarro:** investigation, writing – review and editing. **Javier Miquel‐Miquel:** investigation, writing – review and editing. **Ricardo González‐Pérez:** investigation, writing – review and editing. **Inmaculada Ruiz‐Gonzalez:** investigation, writing – review and editing. **Esther Serra‐Baldrich:** investigation, writing – review and editing. **José Manuel Carrascosa‐Carrillo:** investigation, writing – review and editing. **Francisco Javier Ortiz de Frutos:** investigation, writing – review and editing. **Mercedes Rodríguez‐Serna:** investigation, writing – review and editing. **María Elena Gatica‐Ortega:** investigation, writing – review and editing. **Carmen Paredes Suárez:** investigation, writing – review and editing. **Francisco J. Navarro‐Triviño:** investigation, writing – review and editing. **Pablo Chicharro Manso:** investigation, writing – review and editing. **María Antonia Pastor‐Nieto:** investigation, writing – review and editing. **Marta Andreu Barasoain:** investigation, writing – review and editing. **José‐Juan Pereyra‐Rodríguez:** investigation, writing – review and editing. **Gemma Melé‐Ninot:** investigation, writing – review and editing. **Paloma Sánchez‐Pedreño Guillén:** investigation, writing – review and editing. **Marta Elosua González:** investigation, writing – review and editing. **Miguel Ángel Descalzo:** methodology, formal analysis, writing – review and editing. **Ignacio García‐Doval:** methodology, formal analysis, writing – review and editing. **Raquel Barba Martín:** writing – review and editing. **Leopoldo Borrego:** methodology, data curation, investigation, funding acquisition, supervision, project administration, writing – review and editing.

## Conflicts of Interest

The authors declare no conflicts of interest.

## Supporting information


**Table S1:** Comparison of major studies of Dyshidrotic Eczema and their methodological approach.
**Table S2:** STROBE Checklist—Cross‐sectional DE study (Supporting Information).
**Table S3:** Age distribution of patients with dyshidrotic eczema (DE).
**Table S4:** Clinical relevance of patch test results in patients with DE^†^ and non‐DE hand eczema^‡^. Spanish baseline series 2022 and extended baseline series 2024.
**Table S5:** Association between occupation and DE^†^ versus non‐DE hand eczema^‡^.

## Data Availability

The data that support the findings of this study are available from the corresponding author upon reasonable request and with permission of the Spanish Registry of Research in Contact Dermatitis and Cutaneous Allergy (REIDAC). The data are not publicly available due to privacy and ethical restrictions.
