# Supplementary material for: Epidemiological, Clinical and Allergic Profile of Patients With Dyshidrotic Eczema (Acute and Recurrent Vesicular Dermatitis): Evaluation of the Spanish Registry of Research in Contact Dermatitis and Cutaneous Allergy (REIDAC)
Source: Contact Dermatitis. 2026 May 17;95(3):286–94. doi: 10.1111/cod.70184 (PMC13441355; doi:10.1111/cod.70184)
Supplement: Supplementary file 1 [file COD-95-286-s001.docx]

# **Table S1. Comparison of major studies of Dyshidrotic Eczema and their methodological approach**.

| Study (Year) | N (DE patients) | Setting | Systematic patch testing | Control group (other HE) | Main focus |
| --- | --- | --- | --- | --- | --- |
| Meneghini *et al*. (1979) | Not specified | Single centre | Partial | No | Descriptive clinical series |
| Guillet *et al*. (2007) | 120 | Single centre | Yes | No | Aetiological classification |
| Politiek *et al.* (2020) | 143 | Multicentre | Not systematic | No | Quality of life and disease burden |
| Gladys *et al*. (2018) | 34 932 | Registry-based | Not systematic | No | Epidemiological analysis |
| Present study (2025) | 559 | Multicentre (national) | Yes (100%) | Yes | Clinical and allergological comparison |

**Table S2. STROBE Checklist — Cross-sectional DE study (Supplementary Material)**

| **STROBE Item** | **Manuscript section / Page** | **DE group reporting** | **Non-DE HE reporting** |
| --- | --- | --- | --- |
| 1. Title and abstract | Title + Abstract (p.1,3) | Reported in Abstract | Reported in Abstract |
| 2. Background/rationale | Introduction (p.4) | Reported | Reported |
| 3. Objectives | Introduction (p.4) | Reported | Reported |
| 4. Study design | Methods 2.1 (p.4) | Reported | Reported |
| 5. Setting | Methods 2.2 (p.4) | Reported | Reported |
| 6. Participants | Methods 2.3 (p.5) | Reported | Reported |
| 7. Variables | Methods 2.2 (p.5) | Reported | Reported |
| 8. Data sources/measurement | Methods 2.2 (p.4-5) | Reported | Reported |
| 9. Bias | Discussion 4.3 (p.7) | Reported as limitation | Reported as limitation |
| 10. Study size | Results 3 (p. 6) | Reported (*n*=559) | Reported (*n*=3819) |
| 11. Quantitative variables | Methods 2.5 (p.5-6) | Reported | Reported |
| 12. Statistical methods | Methods 2.5 (p.5-6) | Reported | Reported |
| 13. Flow diagram | Methods 2.3 (p.5)/ Results 3 (p. 6) | Participant numbers reported in text | Participant numbers reported in text |
| 14. Descriptive data | Results (p.6-7) + Table 1-2 | Reported | Reported |
| 15. Outcome data | Results (p.6-7) + Table 3. Figure 1 | Reported | Reported |
| 16. Main results | Results (p.6-7) + Table 3 Figure 1 | Reported | Reported |
| 17. Other analyses | Methods 2.5 (p.5-6)/ Results (p.6-7) + Tables S3–S5 | Exploratory analyses by age, occupation and allergen relevance | Exploratory analyses by age, occupation and allergen relevance |
| 18. Key results summary | Discussion 4.1–4.2 (p.7-9) | Reported | Reported |
| 19. Limitations | Discussion 4.3 (p.9) | Reported | Reported |
| 20. Interpretation | Discussion + Conclusions (p.9-10) | Reported | Reported |
| 21. Generalizability | Discussion (p.7) | Within specialised units | Within specialised units |
| 22. Funding | Methods 2.7 (p.6) | REIDAC hosted by FPS | REIDAC hosted by FPS |
| 23. Ethics | Methods 2.6 (p.6) | Approved CEIC | Approved CEIC |

# **Table S3. Age distribution of patients with dyshidrotic eczema (DE)**

| Age range | N (%) |
| --- | --- |
| Mean age (SD) | 41.6 years (15.4) |
| 0–10 years | 9 (1.6%) |
| 11–20 years | 29 (5.2%) |
| 21–30 years | 109 (19.5%) |
| 31–40 years | 111 (19.9%) |
| 41–50 years | 122 (21.9%) |
| 51–60 years | 108 (19.4%) |
| 61–70 years | 54 (9.7%) |
| 71–80 years | 12 (2.2%) |
| >80 years | 4 (0.7%) |

Data are expressed as number of patients (percentage), unless otherwise indicated. SD, standard deviation.

# **Table S4. Clinical relevance of patch test results in patients with DE^†^ and non-DE hand eczema^‡^. Spanish baseline series 2022 and extended baseline series 2024.**

| **Allergen (ESCD nomenclature; concentration, vehicle)** | **DE total N** | **DE positive N (%)** | **DE current relevance N (%)** | **DE past relevance N (%)** | **DE unknown relevance N (%)** | **Non-DE total N** | **Non-DE positive N (%)** | **Non-DE current relevance N (%)** | **Non-DE past relevance N (%)** | **Non-DE unknown relevance N (%)** |
| --- | --- | --- | --- | --- | --- | --- | --- | --- | --- | --- |
| **Spanish baseline series 2022** | | | | | | | | | | |
| Nickel sulfate 5% petrolatum | 559 | 135 (24.1) | 37 (27.4) | 78 (57.8) | 20 (14.8) | 3808 | 881 (23.1) | 213 (24.2) | 545 (61.9) | 123 (14.0) |
| Lanolin alcohols 30% petrolatum | 559 | 3 (0.5) | 1 (33.3) | 0 (0.00) | 2 (66.7) | 3806 | 14 (0.4) | 10 (71.4) | 1 (7.1) | 3 (21.4) |
| Neomycin sulfate 20% petrolatum | 559 | 1 (0.2) | 0 (0.00) | 0 (0.00) | 1 (100.00) | 3806 | 22 (0.6) | 4 (18.2) | 8 (36.7) | 10 (45.5) |
| Potassium dichromate 0.5% petrolatum | 558 | 18 (3.2) | 5 (27.8) | 4 (22.2) | 9 (50.0) | 3807 | 143 (3.8) | 71 (49.7) | 34 (23.8) | 38 (26.6) |
| Caine mix 10% petrolatum | 465 | 3 (0.6) | 0 (0.00) | 2 (66.7) | 1 (33.3) | 3065 | 18 (0.6) | 1 (5.6) | 6 (33.3) | 11 (61.1) |
| Fragrance mix I 8% petrolatum | 559 | 22 (4.0) | 12 (54.6) | 1 (4.6) | 9 (40.9) | 3807 | 151 (4.0) | 93 (61.4) | 8 (5.3) | 50 (33.1) |
| Colophonium 20% petrolatum | 559 | 5 (0.9) | 1 (20.0) | 2 (40.0) | 2 (40.0) | 3808 | 48 (1.3) | 12 (25.0) | 11 (22.9) | 25 (52.1) |
| Paraben mix 16% petrolatum | 558 | 3 (0.5) | 2 (66.7) | 0 (0.00) | 1 (33.3) | 3806 | 9 (0.2) | 4 (44.4) | 0 (0.00) | 5 (55.6) |
| Balsam of Peru 25% petrolatum | 559 | 18 (3.2) | 5 (27.8) | 4 (22.2) | 9 (50.0) | 3808 | 121 (3.2) | 46 (38.0) | 11 (9.1) | 64 (52.9) |
| Cobalt chloride 1% petrolatum | 559 | 30 (5.4) | 6 (20.0) | 13 (43.3) | 11 (36.7) | 3807 | 185 (4.7) | 54 (29.2) | 64 (34.6) | 67 (36.2) |
| p-tert-Butylphenol formaldehyde resin 1% petrolatum | 559 | 4 (0.7) | 1 (25.0) | 1 (25.0) | 2 (50.0) | 3807 | 41 (1.1) | 7 (17.1) | 4 (9.8) | 30 (73.2) |
| Epoxy resin 1% petrolatum | 559 | 4 (0.7) | 0 (0.0) | 1 (25.0) | 3 (75.0) | 3807 | 34 (0.9) | 22 (64.7) | 3 (8.8) | 9 (26.5) |
| Carba mix 3% petrolatum | 558 | 3 (0.5) | 2 (66.7) | 1 (33.3) | 0 (0.0) | 3787 | 116 (3.1) | 89 (76.7) | 5 (4.3) | 22 (19.0) |
| Black rubber mix / IPPD 0.1% petrolatum | 559 | 3 (0.5) | 1 (33.3) | 0 (0.0) | 2 (66.7) | 3808 | 26 (0.7) | 15 (57.7) | 4 (15.4) | 7 (26.9) |
| MCI/MI 0.02% aqueous | 456 | 21 (4.6) | 14 (66.7) | 1 (4.8) | 6 (28.6) | 3118 | 170 (5.5) | 132 (77.7) | 8 (4.7) | 30 (17.7) |
| Quaternium-15 1% petrolatum | 559 | 7 (1.3) | 4 (57.1) | 0 (0.0) | 3 (42.9) | 3807 | 24 (0.6) | 17 (70.8) | 1 (4.2) | 6 (25.0) |
| p-Phenylenediamine 1% petrolatum | 559 | 15 (2.7) | 3 (20.0) | 7 (46.7) | 5 (33.3) | 3807 | 114 (3.0) | 59 (51.8) | 35 (30.7) | 20 (17.5) |
| Formaldehyde 2% aqueous | 469 | 8 (1.2) | 1 (12.5) | 0 (0.0) | 7 (87.5) | 3240 | 79 (2.4) | 43 (54.4) | 3 (3.8) | 33 (41.8) |
| Mercapto mix 2% petrolatum | 559 | 1 (0.2) | 1 (100.0) | 0 (0.0) | 0 (0.0) | 3807 | 13 (0.3) | 11 (84.6) | 0 (0.0) | 2 (15.4) |
| Thiuram mix 1% petrolatum | 558 | 3 (0.5) | 2 (66.7) | 1 (33.3) | 0 (0.0) | 3807 | 105 (2.8) | 98 (93.3) | 2 (1.9) | 5 (4.8) |
| Diazolidinyl urea 2% petrolatum | 559 | 2 (0.4) | 1 (50.0) | 0 (0.0) | 1 (50.0) | 3807 | 13 (0.3) | 7 (53.9) | 0 (0.0) | 6 (46.2) |
| Tixocortol pivalate 1% petrolatum | 558 | 2 (0.4) | 2 (100.0) | 0 (0.0) | 0 (0.0) | 3806 | 7 (0.2) | 3 (42.9) | 1 (14.3) | 3 (42.9) |
| Imidazolidinyl urea 2% petrolatum | 559 | 1 (0.2) | 1 (100.0) | 0 (0.0) | 0 (0.0) | 3807 | 12 (0.3) | 4 (33.3) | 1 (8.3) | 7 (58.3) |
| Budesonide 0.01% petrolatum | 559 | 1 (0.2) | 1 (100.0) | 0 (0.0) | 0 (0.0) | 3806 | 15 (0.4) | 5 (33.3) | 0 (0.0) | 10 (66.7) |
| Mercaptobenzothiazole 2% petrolatum | 559 | 2 (0.4) | 0 (0.0) | 0 (0.0) | 2 (100.0) | 3807 | 16 (0.4) | 13 (81.3) | 0 (0.0) | 3 (18.8) |
| Methylisothiazolinone 0.2% aqueous | 505 | 39 (7.7) | 29 (74.4) | 3 (7.7) | 7 (18.0) | 3365 | 274 (8.1) | 231 (84.3) | 16 (5.8) | 27 (9.9) |
| Fragrance mix II 14% petrolatum | 505 | 10 (2.0) | 7 (70.0) | 0 (0.0) | 3 (30.0) | 3371 | 100 (3.0) | 59 (59.0) | 6 (6.0) | 35 (35.0) |
| 2-Hydroxyethyl methacrylate (2-HEMA) 2% petrolatum | 399 | 12 (3.0) | 9 (75.0) | 2 (16.7) | 1 (8.3) | 2655 | 302 (11.4) | 245 (81.1) | 13 (4.3) | 44 (14.6) |
| Textile dye mix 6.6% petrolatum | 391 | 4 (1.0) | 0 (0.0) | 0 (0.0) | 4 (100.0) | 2617 | 65 (2.5) | 24 (36.9) | 3 (4.6) | 38 (58.5) |
| Hydroperoxides of linalool 0.5% petrolatum | 402 | 10 (2.5) | 5 (5000) | 0 (0.00) | 5 (50.0) | 2665 | 122 (4.6) | 85 (69.7) | 2 (1.6) | 35 (28.7) |
| Hydroperoxides of limonene 0.2% petrolatum | 404 | 9 (2.2) | 5 (55.6) | 0 (0.0) | 4 (44.4) | 2671 | 97 (3.6) | 60 (61.9) | 2 (2.1) | 35 (36.1) |
| **Extended baseline series 2024** | | | | | | | | | | |
| Methyldibromo glutaronitrile 0.1% petrolatum | 331 | 12 (3.6) | 1 (8.3) | 0 (0.0) | 11 (91.7) | 2356 | 88 (3.7) | 11 (12.5) | 3 (3.4) | 74 (84.1) |
| Sesquiterpene lactone mix 0.1% petrolatum | 411 | 0 (0.0) | 0 () | 0 () | 0 () | 2735 | 11 (0.4) | 3 (27.3) | 0 (0.0) | 8 (72.7) |
| Hydroxyisohexyl 3-cyclohexene carboxaldehyde (Lyral) 5% petrolatum | 412 | 6 (1.5) | 5 (83.3) | 0 (0.0) | 1 (16.7) | 2729 | 11 (0.4) | 6 (54.6) | 3 (27.3) | 2 (18.2) |
| Propolis 10% petrolatum | 264 | 12 (4.6) | 1 (8.3) | 1 (8.33) | 10 (83.3) | 1801 | 109 (6.1) | 23 (21.1) | 8 (7.3) | 78 (71.6) |
| Sodium disulfite 1% petrolatum | 264 | 4 (1.5) | 1 (25.0) | 0 (0.0) | 3 (75.0) | 1804 | 35 (1.9) | 6 (17.1) | 0 (0.0) | 29 (82.9) |
| 2-bromo-2-nitropropane-1,3-diol (Bronopol) 0.5% petrolatum | 261 | 0 (0.0) | 0 () | 0 () | 0 () | 1790 | 12 (0.7) | 8 (66.7) | 0 (0.0) | 4 (33.3) |
| Compositae mix II 5% petrolatum | 88 | 0 (0.0) | 0 () | 0 () | 0 () | 648 | 6 (0.9) | 4 (66.7) | 0 (0.0) | 2 (33.3) |
| Benzisothiazolinone 0.1% petrolatum | 264 | 6 (2.3) | 4 (66.7) | 0 (0.0) | 2 (33.3) | 1809 | 110 (6.1) | 70 (63.6) | 2 (1.8) | 38 (34.6) |
| Octylisothiazolinone 0.1% petrolatum | 264 | 2 (0.7) | 1 (50.0) | 0 (0.0) | 1 (50.0) | 1801 | 19 (1.1) | 11 (57.9) | 0 (0.0) | 8 (42.1) |
| Decyl glucoside 5% petrolatum | 264 | 1 (0.4) | 1 (100.0) | 0 (0.0) | 0 (0.0) | 1802 | 6 (0.3) | 3 (50.0) | 0 (0.0) | 3 (50.0) |
| Sorbitan sesquioleate 20% petrolatum | 117 | 0 (0.0) | 0 () | 0 () | 0 () | 705 | 4 (0.6) | 2 (50.0) | 0 (0.0) | 2 (500) |
| Sorbitan monooleate (Span 80) 5% petrolatum | 117 | 0 (0.0) | 0 () | 0 () | 0 () | 700 | 2 (0.3) | 1 (50.0) | 0 (0.0) | 1 (50.0) |
| Hydroperoxides of linalool 0.5% petrolatum | 400 | 5 (1.3) | 2 (40.0) | 0 (0.0) | 3 (60.0) | 2613 | 84 (3.2) | 56 (66.7) | 2 (2.4) | 26 (31.0) |
| Hydroperoxides of limonene 0.2% petrolatum | 398 | 4 (1.0) | 1 (25.0) | 0 (0.0) | 3 (75.0) | 2605 | 65 (2.5) | 40 (61.5) | 2 (3.1) | 23 (35.4) |

Data are expressed as N (%). Allergen concentrations and vehicles follow ESCD/Contact Dermatitis nomenclature.

^†^DE: dyshidrotic ezcema, ^‡^Non -DE: non- dyshidrotic ezcema.

# **Table S5. Association between occupation and DE^†^ vs non-DE hand eczema^‡^.**

| Occupation | DE N (%) | Non-DE N (%) | OR | 95% CI | p value |
| --- | --- | --- | --- | --- | --- |
| Healthcare workers | 58 (11) | 456 (12) | 0.86 | [0.64, 1.15] | 0.2982 |
| Administrative workers | 72 (13) | 350 (9) | 1.47 | [1.12, 1.93] | 0.0051 |
| Services | 30 (5) | 207 (5) | 0.99 | [0.67, 1.47] | 0.9765 |
| Waiters | 22 (4) | 155 (4) | 0.97 | [0.62, 1.53] | 0.9056 |
| Hairdressing / Aesthetics | 14 (3) | 312 (8) | 0.29 | [0.17, 0.50] | <0.0001 |
| Food processing | 11 (2) | 125 (3) | 0.60 | [0.32, 1.11] | 0.1032 |
| General cleaning | 26 (5) | 211 (6) | 0.84 | [0.55, 1.27] | 0.4062 |
| Housewife/househusband | 41 (7) | 248 (7) | 1.15 | [0.81, 1.61] | 0.4388 |
| Students | 58 (11) | 286 (8) | 1.44 | [1.07, 1.94] | 0.0168 |
| Retired | 37 (7) | 294 (8) | 0.85 | [0.60, 1.22] | 0.3814 |
| Other occupations | 180 (33) | 1123 (30) | 1.15 | [0.95, 1.39] | 0.1562 |

Data are expressed as number of patients (percentage). OR, odds ratio; CI, confidence interval.

^†^DE: dyshidrotic ezcema, ^‡^ Non -DE: non- dyshidrotic ezcema.
